# Reply to Cione et al. Comment on “Moshirfar et al. Accuracy of Six Intraocular Lens Power Calculations in Eyes with Axial Lengths Greater than 28.0 mm. *J. Clin. Med.* 2022, *11*, 5947”

**DOI:** 10.3390/jcm12093268

**Published:** 2023-05-04

**Authors:** Majid Moshirfar, Kathryn M. Durnford, Jenna L. Jensen, Daniel P. Beesley, Telyn S. Peterson, Ines M. Darquea, Yasmyne Castillo Ronquillo, Phillip C. Hoopes

**Affiliations:** 1 Hoopes Vision, HDR Research Center, Draper, UT 84020, USA; 2 John A. Moran Eye Center, Department of Ophthalmology and Visual Sciences, Salt Lake City, UT 84132, USA; 3 Utah Lions Eye Bank, Murray, UT 84107, USA; 4 School of Medicine, University of Utah, Salt Lake City, UT 84132, USA; 5 Brigham Young University, Provo, UT 84602, USA; 6 College of Osteopathic Medicine, Rocky Vista University, Ivins, UT 80112, USA

We thank Cioni et al. for their suggestions and comments [1]. The limitations of a retrospective study using real-world data are ever-present. Moreover, the statistical analysis of these studies presents challenges.

1. We agree that using the most recent adjustments is optimal. We expect that using the updated adjustments will have minimal effects on our findings. Indeed, the difference, by calculating 38 data points, shows an average absolute difference of 0.031 mm (SRKT) and 0.036 mm (Holladay) in axial lengths between the 2011 and 2018 formulas. This suggests that the difference results in clinically non-significant changes. The IOL power results in a change of 1/100 to 1/10 of a Diopter, which is clinically not significant. (Diopters of IOLs are in 0.5 D increments.)
2. We used the Wilcoxon test as a sequential paired method, as shown in Table 3. This is an acceptable alternative analysis. We used the Friedman test for our previous IOL formula statistical analysis. Please see Rosen et al [2]. Note that we used both Wilcoxon and Friedman in this previously published manuscript, but this study involved an intervention—refractive surgery. One recent paper with an intervention (RK) used the Wilcoxon tests for pairwise comparisons with Bonferroni correction after the Friedman tests were significant. This paper compared three formulas [3].
3. A. We did mention the confounding variable, but since this is purely a mathematical calculation, the risk of confounding is minimal compared to the efficiency of using bilateral data in an uncommon phenomenon. The correlation of bilateral data, we believe, is more affected by interventions (for example, drugs and laser treatments where endpoints or outcomes are measured) or disease states rather than diagnostic tests which yield pure numbers [4].
  The discussion in this article is about applying GEE and other methods of analyzing correlated data in interventional or outcome studies.
  B. While we agree that sample size calculation can be performed, it is not imperative for the real-world mathematical data of IOL formulas analyzed retrospectively for accuracy. For prospective interventional studies, computing sample size is the rule. For rare diseases, this is challenging. High myopia in a predominantly Caucasian population is not too common. A multicenter prospective or retrospective study would be ideal and yield more samples to analyze.
4. We used ULIB because studies in the United States use this platform or database, and furthermore, Hoffer and Savini also recommended it. Thank you for the suggestion; we will look into this European database in our future studies and compare it with ULIB.
5. Thank you for your comment. Contrary to your assertion on the use of Python, this suggestion is indeed by Hoffer and Savini, in the fifth paragraph on the topic of constant optimization.
*“Optimization of the unpublished formulas (e.g., Barrett Universal II, EVO, Kane, and Olsen) is not possible using only Excel. Hence, 2 possibilities exist: the first is to contact their authors and ask them to do it for you. The second is using specific computer programming languages able to extract data automatically from any database (e.g., Python Software Foundation, Wilmington, DE), enter them into the formula website, and generate a new database containing the predicted refraction for each eye. Regarding the Holladay 2 formula, which is also unpublished, it is possible to perform optimization using the Holladay IOL Consultant Software & Surgical Outcomes Assessment (www.hicsoap.com).”*
  Therefore, on page 3 of our article, we stated,
  “For the remaining four unpublished formulas, ULIB IOL constants and biometrics were input into the online calculators via Python (Python Software Foundation, Wilmington, DE, USA) as recommended by Hoffer and Savini.”[5]
  We are confident that the statistical tests in this study are adequate. Nevertheless, we will apply some of your suggestions in our future studies when warranted.

## Data Availability

The data presented are available upon request to the corresponding author. The data are not publicly available due to patient privacy.
